# Hints on the Treatment of Constipation

**Published:** 1876-03

**Authors:** 


### HINTS ON THE TREATMENT OF CONSTIPATION.

Habitual constipation is not a trivial affection. Its management requires much care and perseverance on the part of both physician and patient. Its causes are numerous, and should be diligently sought for, if we expect to manage a case with any degree of success. The constipation of the young is the result of dryness and solidification of the fæcal matter from active absorption in the small intestines, and without treatment, under ordinary circumstances, would cease after a few years. By improper treatment, it often becomes a serious affection; and, at the time when nature should afford relief, we find many suffering from obstinate constipation, and the long train of symptoms incident thereto. In the treatment of these, do away with all cathartics and laxatives. The giving of aperients by the mouth for a very local affection confined to the opposite extremity of the intestinal tube, besides being a circuitous measure, is moreover attended with inconvenience and disadvantages. Commonly, we can accomplish all that is desirable by proper attention to diet. The food should be pultaceous and herbaceous. A porridge once a day of barley and oat meal will often be sufficient to regulate the bowels. Ripe fruit should be used with little restraint, and lemonade or cider drank freely. Should this system of diet fail to remove the affection, the next thing to be tried in addition is injections. A simple one of considerable volume of tepid water should first be employed; this failing, it can be medicated with castor or olive oil or turpentine.

In cases of constipation from indolence of the bowels we find especial indications for belladonna, nux vomica, and astringent injections. It is now generally believed that belladonna brings about increased peristaltic action. The cause of this increased action may be direct stimulation of the muscular coat by atropia carried to it with the blood, but other causes have been suggested which seem worthy of consideration. When this drug is administered in small medicinal doses it causes a remarkable dryness of the throat and tongue, difficulty in, yet constant effort at, deglutition. The changes in the act of micturition are remarkable and noteworthy. This is often hurried and frequent, sometimes interrupted, and occasionally there is slight strangury. In the throat the mucous secretion is obviously checked, the membrane is seen to be dry, and its surface is rendered more susceptible of irritation; hence the constant efforts at deglutition. I believe the effect of the drug on the mucous membranes to be of the same nature; and in the bladder this arrest of mucous secretion results in irregular and frequent micturition. According to the above view, its action on the bowels is easily explained. The mucous secretion being checked, the irritation caused by the contents of the intestinal canal, when its surface is thus unprotected, produces more prompt and vigorous contractile action.

Nux vomica acts as a stimulant to the motor nerves, and is especially indicated in those cases where there is reason to suspect a general want of tone in the bowels in consequence of long-continued distention. By acting as a tonic to the muscular coat of the intestines, it increases very sensibly the activity of purgative medicines. An apeirent scarcely sufficient by itself to produce a single evacuation, when combined with the extract of nux vomica, causes active purgation, and this is not followed by that reaction that characterizes purgative medicines when given as such, but on the contrary, the improved action of the bowels is, comparatively speaking, sustained. I have used the following with excellent results:

R.—Ext. Nux Vomica............................gr. v.
Ext. Colocynth Comp...........................
Pulv. Aloes. aa.................................gr. xv.
Ext. Belladonna..................................gr. vij.
Ferri Sulphas (exsis)..........................gr. xv.
M.

Fiat. pil. No. xx. One pill to be taken at bed time. Warm water injections should never be given in such cases. They are injurious, because, as the muscular fibers are in a state of atony, they are thereby lengthened, softened, and deprived of their contractile power. Injections of cold water may be given with advantage, as they rouse the sensibility and contractile power of the intestine. In some cases I have seen good results from astringent injections. In some persons that have long suffered from constipation, particularly females, the rectum forms above the sphincter a pouch sometimes of considerable size, in consequence of distention from accumulated fæces to which the coats of the bowels have been subjected. Astringint injections into the rectum cause corrugation of the the muscular fibres of the bowels, which by corrugation become shorter, and thus diminish the enlargement of the cul-de-sac spoken of. These injections may be used with advantage when there is reason to suspect an abnormal dilatation of the lower portion of the rectum; for instance, in constipation from the presence of a mechanical obstacle at the anus, caused by hæmorrhoidal tumors, swellings of a venereal, cancerous character, or contraction of the sphincter with or without fissure. These injections are, moreover, suitable, for the same reason, to females in whom constipation exists along with engorgement or retroversion of the uterus, and all persons who, having their bowels relieved only once in eight or ten days, void, after painful efforts which can be compared to nothing but a sort of parturition, an enormous mass of hardened and dry fæces. The ingredients of these injections may be infinitely varied; they may be composed of oak bark, catechu, alum, etc. Whatever plan of treatment is adopted, it is of great importance that the diet of the patient should be regulated, and all should be instructed to go to stool at certain hours each day, whether they felt called by nature to do so or not.—*Indiana Journal of Medicine.*

